# 

**Published:** 1896-03-28

**Authors:** 


**CADBURY'S COCOA**

"The standard of highest purity at present attainable."—*Lancet.*

**CADBURY'S COCOA**
NO ALKALIES USED.

### THE HOSPITAL

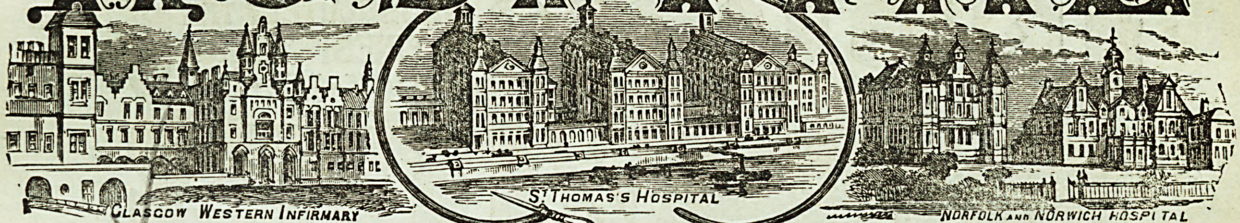

No. 496. Vol. XIX.

SATURDAY, MARCH 28TH, 1896.

**WITH EXTRA NURSING SUPPLEMENT.**

[Registered at the General Post Office as a Newspaper for transmission in the United Kingdom and Abroad.]

**PRICE TWOPENCE.**

Per Annum, 10s. 6d., post free.

Monthly Parts, 6d.; post free, 8d.

Ditto per Annum, 8s.6d., post free.

